# Schick Test

**Published:** 1915-11

**Authors:** 


**Schick Test.** 1/10 C. C. of diphtheria toxin or 2/10 C. C. of 1:1000 or, if the first test is doubtful, 2/10 C. C. of 1:5000, is injected intracutaneously in the forearm. If an erythema appears within 24-48 hours, it is considered that the organism contains no protective bodies against diphtheria. Jerome Zuckerman of N. Y., N. Y. Med. Jour., Oct. 16, found the test positive (immunization indicated) in 20 of 52 children under 1 year; in 67 of 104 children betweetn 1 and 2; in 59 of 124 children between 4 and 6. Schick found positive reactions in 7% of children under 1 year and in 53% of those between 1 and 5 years; Kolmer, 12% and 54%; Park 40% and 60%; Zuckermann 38.5% and 49%. Various other tables are given, as of time of return of positive reaction after injection.

